# Susceptibility of Diabetic Patients to COVID-19 Infections: Clinico-Hematological and Complications Analysis

**DOI:** 10.3390/vaccines11030561

**Published:** 2023-03-01

**Authors:** Banan Atwah, Mohammad Shahid Iqbal, Saeed Kabrah, Ahmed Kabrah, Saad Alghamdi, Aisha Tabassum, Mohammed A. Baghdadi, Hissah Alzahrani

**Affiliations:** 1 Laboratory Medicine Department, Faculty of Applied Medical Sciences, Umm Al-Qura University, Makkah 21955, Saudi Arabia; 2 Research Center, King Faisal Specialist Hospital and Research Centre (KFSH&RC), Jeddah 23431, Saudi Arabia; 3 Mathematical Sciences Department, Faculty of Applied Sciences, Umm Al-Qura University, Makkah 21955, Saudi Arabia

**Keywords:** COVID-19, diabetics, Saudi Arabia, susceptibility

## Abstract

Background: Coronavirus disease 2019 has become a global health threat resulting in a catastrophic spread and more than 3.8 million deaths worldwide. It has been suggested that there is a negative influence of diabetes mellites (DM), which is a complex chronic disease, on COVID-19 severe outcomes. Other factors in diabetic patients may also contribute to COVID-19 disease outcomes, such as older age, obesity, hyperglycaemia, hypertension, and other chronic conditions. Methods: A cohort study was conducted on the demographics, clinical information, and laboratory findings of the hospitalised COVID-19 with DM and non-DM patients were obtained from the medical records in King Faisal Specialist Hospital and Research Centre, Saudi Arabia. Results: Among the study population, 108 patients had DM, and 433 were non-DM patients. Patients with DM were more likely to present symptoms such as fever (50.48%), anorexia (19.51%), dry cough (47.96%), shortness of breath (35.29%), chest pain (16.49%), and other symptoms. There was a significant decrease in the mean of haematological and biochemical parameters, such as haemoglobin, calcium, and alkaline phosphate in people with diabetes compared to non-diabetics and a considerable increase in other parameters, such as glucose, potassium, and cardiac troponin. Conclusions: According to the findings of this study, patients who have diabetes have a greater risk of developing more severe symptoms associated with COVID-19 disease. This could result in more patients being admitted to the intensive care unit as well as higher mortality rates.

## 1. Introduction

The first severe acute respiratory syndrome (SARS)-associated coronavirus was discovered in China in February 2003, during the SARS outbreak, which then spread to four other countries [1]. SARS can be transmitted from an infected person via aerosols or droplets, which can be inhaled directly or indirectly through contact with affected surfaces [2]. At the end of December 2019, there was evidence of pneumonia cases caused by an unknown causative agent in China (Wuhan city) [3]. It was confirmed as the 2019 novel Coronavirus on 7 January 2020, and is now known as Severe Acute Respiratory Syndrome Coronavirus 2 (SARS-CoV-2) [4]. On 6 February 2020, the Saudi government implemented early measures and restrictions to prevent the introduction and spread of SARS-CoV-2 in the country. The World Health Organization (WHO) declared coronavirus disease 2019 on 11 February 2020 as the name of the epidemic disease caused by SARS-CoV-2 (COVID-19) [4]. On 2 March 2020, the first case of COVID-19 was reported in the Kingdom of Saudi Arabia (KSA) [5]. Since then, despite the early restrictions, more cases have been reported, with 8200 cases and 92 deaths recorded by 18 April 2020.

Although SARS-CoV-2 mostly affects the lungs, it can also impact the brain, heart, and digestive tract. It has been noted that 75% of COVID-19 patients who are hospitalised have at least one COVID-19-linked comorbidity. Additionally, SARS-CoV-2 causes consequences related to hypercoagulability such as gangrene, stroke, pulmonary embolism, and other related concerns [6] In extremely or critically ill individuals with comorbidities, neurological problems are common [7]. The most common cardiovascular complications described in COVID-19 patients are increased risk for myocardial infarction, fulminant myocarditis rapidly evolving with depressed systolic left ventricle function, arrhythmias, venous thromboembolism, and cardiomyopathies mimicking STEMI presentations [8]. The risk factors associated with COVID-19 infection prognosis and mortality have been documented. They include older age and underlying medical conditions such as hypertension, diabetes mellitus (DM), and coronary heart disease [9,10,11]. Impairments in insulin secretion/function and glucose metabolism are at the heart of type II diabetes mellitus (T2DM), a chronic metabolic disorder with a worldwide impact [12]. Alguwaihes A et al. suggested that the number of hospitalised COVID-19 positive diabetic patients is greater than the number of non-diabetic patients by 2:1 [13]. Similarly, the mortality rates are higher in COVID-19 patients with DM than in patients without DM, which may be due to severe clinical symptoms in the diabetic patients [13,14]. However, it has been demonstrated that DM is not the only factor associated with hospital deaths in COVID-19-infected patients [15]. Although DM is more frequent in COVID-19 patients, a study in the United States demonstrated a lack of evidence of a direct link between diabetes in these patients and the mortality [16]. Therefore, other potential factors, such as other chronic diseases and cardiometabolic dysfunctions in diabetic patients, may contribute to a worse COVID-19 outcome [17,18]. It was also suggested that severe vitamin D deficiency, elevated creatinine, smoking, and the use of β-blockers might also influence COVID-19 severity and mortality rates [13]. The haematological parameters in COVID-19-infected patients with and without type 2 DM were investigated [19]. It was demonstrated that white blood cells (WBCs) and neutrophil counts in the diabetic patient group were more significant than in the non-diabetic group. In contrast, there was no significant difference in the red blood cell (RBC) count between the two groups [19]. In this present study, we focused on exploring the association of DM and COVID-19 infection in terms of clinical presentation, laboratory parameters, and its effect on the disease outcome.

## 2. Materials and Methods

### 2.1. Study Design and Study Population

A retrospective cohort analysis was conducted on hospitalised confirmed COVID-19 patients admitted to King Faisal Specialist Hospital and Research Centre (KFSH&RC) Riyadh and Jeddah, Saudi Arabia, between 1 March 2020 and 31 March 2021. All confirmed COVID-19 patients with complete laboratory investigation are included in the current study. 

### 2.2. Data Collection 

Patients’ data were obtained from the information system for patients diagnosed according to the WHO COVID-19 guidelines with a positive nasal and pharyngeal swab real-time reverse transcriptase-polymerase chain reaction. Data contained demographic information, clinical information, and laboratory findings.

### 2.3. Statistical Analysis

The data analysis was conducted using SAS programming language. Descriptive analysis of frequency, percentage, and mean with standard deviation were performed for categorical and continuous variables. Following tests for normality (Shapiro–Wilk test), variables that did not fulfil the normality assumption were compared using the Wilcoxon test. Chi-squared and Fisher tests were performed to evaluate the differences in the prevalence of categorical variables. A *p*-value of 0.05 was used to indicate a statistically significant result.

## 3. Results

### 3.1. Demographic and Clinical Data

The current study included 541 patients, out of which, 108 (19.96%) were diabetic and 433 (80.04%) were non-diabetic. There were 252 (47.01%) female and 289 (53.41%) male patients. The mean ages ± SD for the DM and non-DM groups were 60.3 ± 14.23 and 37.4 ± 18.8 years respectively. The age variation was significant between the two groups as diabetics were much older than the non-diabetics (*p* = <0.0001). The body mass index was significantly higher in diabetics in contrast to non-diabetics (*p* = 0.0002). As this was a hospital-based study, the patients included corresponded to categories 3 to 7 of the WHO ordinal scale. Based on the available data, we could not differentiate them further separately under each scale of the WHO. On admission, when compared with the non-diabetics, the diabetics were more likely to present with symptoms including fever (50.48%, *p* = <0.000*), anorexia (19.51%, *p* = 0.002), dry cough (47.96%, *p* = 0.024), shortness of breath (35.29%, *p* = 0.002), chest pain (16.49%, *p* = 0.010), haemoptysis (4.60%, *p* = 0.023), confusion (6.98%, *p* = 0.000*), loss of consciousness (4.30%, *p* = 0.024), vomiting (16.67%, *p* = 0.035), and diarrhoea (25.77%, *p* = 0.012) as shown in Table 1. 

### 3.2. Haematological and Biochemical Findings

Table 2 describes the laboratory data from DM and non-DM cohorts. In diabetic patients, when compared with the non-diabetics, there was a statistical significant decreases in the mean haemoglobin (*p* = <0.0001), haematocrit (*p* = <0.0001), serum sodium (*p* = <0.0001), bicarbonate (*p* = 0.02), calcium (*p* = <0.0001), and alkaline phosphate (*p* = 0.06) values, whereas there was a significant increase in prothrombin time (*p* = 0.09), partial thromboplastin time (*p* = 0.088), serum urea (*p* = <0.0001), creatinine (*p* = <0.0001), glucose (*p* = <0.0001), alanine aminotransferase (*p* = 0.0023), aspartate amino transferase (*p* = <0.0001), PO2 (*p* = 0.0031), PO2/FiO2 (*p* = 0.0587), lactate (*p* = 0.034), ferritin (*p* = <0.0001), potassium (*p* = 0.0061), total bilirubin (*p* = 0.0014), creatine kinase (*p* = 0.0169), and cardiac troponin (*p* = <0.0001). There was no significant variation for other lab variables between the two groups.

Out of the 108 diabetics, 81 (75%) developed severe disease and complications, whereas, among the non-diabetics, 208 (48%) developed severe disease. A significant finding was increased ICU admissions and the necessity for mechanical ventilation among the DM group compared to the non-DM group. (Table 3). Furthermore, among the diabetics, 10 (3.5%) developed renal failure and 11 (3.9%) developed sepsis. In contrast, only 6 (2.1%) and 6 (2.1%) for non-diabetics, respectively, developed these symptoms, indicating that the clinical outcome in diabetic COVID-19 patients is worse in contrast to that of non-diabetic COVID-19 patients. Mortality was significantly high among the diabetic cohort when compared to non-diabetics (35.18% vs. 20.6%).

## 4. Discussion

COVID-19 and type 2 diabetes mellitus (T2DM) are inversely correlated. Diabetes with poor control makes COVID-19 more severe and is linked to higher morbidity and mortality. Additionally, the COVID-19 pandemic has led to poor diabetes control, the advancement of prediabetes to diabetes, an increase in the number of newly diagnosed cases of the disease, and a surge in corticosteroid-induced diabetes [20]. In this retrospective study, we analysed the clinical and laboratory data from 541 COVID-19 patients, including 108 cases of type 2 diabetes and 433 non-diabetic cases. The clinical and laboratory characteristics of COVID-19 in diabetic patients have been reported in numerous other studies as well [13,19,21,22]. In our study, there was a statistically significant age difference between the two groups. Patients in the diabetic group made up a disproportionately high percentage of the elderly, whereas those in the non-diabetic group were generally younger. A similar result was also reported by a Saudi Arabian study [13]. A study from Iran reported that the median age of COVID-19 patients with diabetes was 59 years, while the median age of non-diabetic patients was 37 years, similar to our findings [23].

Diabetes is a chronic disease of the elderly, which explains why the average age of COVID-19 patients with diabetes is higher. Other studies have demonstrated that old age is a major risk factor influencing the prognosis of COVID-19 [9,24]. The prevalence of confirmed diabetes among hospitalised COVID-19 patients was 19.96% in this study. The pooled prevalence of diabetes among hospitalised COVID-19 patients was 14.34%, according to a meta-analysis of 83 observational studies [21]. Multiple reports have shown that people with diabetes are more likely than those without diabetes to develop a life-threatening case of COVID-19 infection [25]. Previous reports indicated an increased morbidity and mortality in diabetes patients infected with Streptococcus pneumonia and influenza virus. [26].

In our study, there was a significant difference between the two groups in terms of the presenting symptoms. Compared to non-diabetic patients, diabetic patients were more likely to present with fever, dry cough, shortness of breath, chest pain, anorexia, haemoptysis, confusion, loss of consciousness, vomiting, and diarrhoea. Similar to what we found, a study from Iran found that people with diabetes most often complain of fever, shortness of breath, and cough. On the other hand, people without diabetes were more likely to have chest pain and a sore throat [23]. Many other studies have also found that the groups have similar clinical signs [27,28].

In contrast to the non-DM group, we observed a significantly higher incidence of diarrhoea in the DM patients. It is well known that diabetic complications such as neurodegeneration, which can cause sympathetic and vagal nerve dysfunction and increase gastrointestinal peristalsis, resulting in diarrhoea or constipation, are not uncommon [19]. High blood glucose levels can disrupt normal intestinal bacterial flora and promote the growth of harmful bacterial flora, both of which can lead to diarrhoea [29]. Therefore, people with diabetes caused by COVID-19 should focus on preserving a healthy balance of bacteria in their intestinal tract.

We found a significant difference in the basal metabolic index (BMI) between groups, with people with diabetes being more likely to be obese. Obese people were more likely to develop serious disease and complications [30].

Anaemia is a common complication of diabetes, which is a chronic condition. In our research, we found that both diabetics and non-diabetics had significantly lower haemoglobin and haematocrit levels than healthy controls. People with diabetes had significantly lower levels of sodium, bicarbonate, and calcium, and higher serum potassium, compared to the control group in our study. Prothrombin and activated partial thromboplastin times were significantly elevated in the diabetes group, as were liver enzymes and cardiac-specific markers such as troponin and creatine kinase. In a separate study, individuals with diabetes and COVID-19 were found to have low serum sodium and elevated aPTT [24].

Some biochemical and haematological alterations in type 2 diabetes mellitus patients differ from those in healthy persons. These metrics should be closely followed up on and supervised in diabetic individuals. A comparison of non-COVID-19 diabetics and non-diabetics revealed that the T2DM group had higher mean values of SGPT, alkaline phosphatase, urea, serum creatinine, total cholesterol, triglycerides, and LDL than the control group did. When compared to the control group, the mean values of haemoglobin, RBC, MCV, MCHC, and MCH in the T2DM group were significantly lower. The T2DM group had a substantially greater mean white blood cell count and differential white blood cell count than the control group. The mean neutrophil/lymphocyte ratio and platelet/lymphocyte ratio in the T2DM group were not statistically different from the control group [31]. The rapid and severe decline in metabolic activity in diabetic patients is explained by a direct effect of SARS-CoV-2 on the function and survival of beta cells [18]. Diabetics had impaired innate and adaptive immunity, which was characterised by a persistent, low-grade inflammatory state that abruptly changed their systemic metabolic state [22,30].

Many tissues contained ACE2, the COVID-19 binding receptor in host cells. The respiratory system, particularly the lung, cardiac tissue, and the gastrointestinal and renal systems, particularly proximal tubular cells, have the highest levels of transcripts [32,33]. Diabetes patients have higher levels of ACE2, which facilitates virus uptake and increases the risk of disease severity [34,35]. SARS-CoV-2 binds to ACE2 receptors, which are expressed in pancreatic tissue, and beta cells in particular, leading to a decrease in insulin secretory capacity of beta cells. Thrombosis is a well-known complication of severe COVID-19, and it may be made worse by stress and cytokine storm, which may cause diabetic ketoacidosis or hyperglycaemic hyperosmolar syndrome [18]. Increased coagulation and thrombotic and inflammatory events in COVID-19 and increased coagulability in diabetes may be a mechanism linking the severity of COVID-19 to diabetes [32].

There have also been reports of endothelial cell dysfunction, compromised platelet function, and coagulation disturbance leading to atherosclerosis and cardiovascular complications [36]. With type 2 diabetes, mildly elevated liver enzymes are frequently observed. Along with insulin resistance, metabolic syndrome, and type 2 diabetes, increased activity of liver enzymes such as AST, ALT, and GGT that indicate liver injury was also linked to these conditions [37,38]. Abnormalities in insulin-sensitive tissues, such as the liver, in terms of triglyceride storage and lipolysis were early indicators of insulin resistance. A high concentration of free fatty acids is a direct toxicant to hepatocytes, which is why insulin-resistant patients tend to have high levels of this compound. Hepatocyte injury is associated with elevated levels of proinflammatory cytokines, a feature shared by diabetes mellitus and COVID-19 [38]. The activation of CD4+ T cell differentiation through Th1 and Th2 cells, as well as the dysfunction of Th17 cells and T regulatory cells, which affect the balance of pro- and anti-inflammation, are all potential pathways that diabetes may follow. Immune system imbalance may also lead to the secretion of inflammatory cytokines [39].

In our study, diabetic patients outnumbered non-diabetics in the severe illness cohort by a ratio of 2:1 (76% vs. 48%). According to other studies, hyperglycaemia patients with COVID-19 had a higher incidence of severe COVID-19 than normoglycemic individuals [40,41]. A previous Saudi Arabian study found comparable results [13]. According to the same survey, COVID-19 patients with diabetes have a higher mortality rate than patients without diabetes. However, other factors such as advanced age, congestive heart failure, smoking, beta blocker use, prevalence of bilateral lung infiltrates, elevated creatinine, and serum vitamin D deficiency, are more accurate predictors of fatal outcome [13]. According to another study, 26.8% of the elderly COVID-19 patients with an increased risk of death were diabetic [42]. In a recent investigation, the function of circulating monocyte subsets and NK cells in the genesis and severity of COVID-19 in diabetics was reported. NK cells exhibit anti-SARS-CoV-2 activity but are diminished functionally in severe COVID-19. Furthermore, since COVID-19 sufferers’ NK cells showed poor anti-fibrotic activity, NK cell dysfunction may play a role in the progression of the disease to fibrotic lung disease. Long-lasting NK cell dysfunction was brought on by a heightened IFN-α response and associated with an unfavourable disease trajectory, suggesting NK cells’ involvement in the immunopathogenesis of COVID-19 [43]. Pulmonary fibrosis, one of the aftereffects of SARS-CoV-2 infection that can cause chronic dyspnoea and necessitate oxygen supplementation after COVID-19, is more prevalent in persons with poorly controlled diabetes. It is also realistic to say that post-COVID-19, an already-existing low-grade inflammatory condition, such as that found in T2DM, may become worse and continue to be at a high level, which may result in a number of symptoms [20].

In our study, there was a big difference between the DM group and the non-DM group when it came to being admitted to the ICU and needing mechanical ventilation. In the DM group it was 17.9%, compared to 12.3% for the non-DM group. Others have reported finding the same results [44]. According to a study from China, diabetic patients with COVID-19 were more likely to die if they were elderly males with hypertension and cardiovascular disease who also presented with shortness of breath. Patients with diabetes were more susceptible to complications, had a greater proportion of ICU admissions, and had a high mortality rate [15].

Previous studies indicate that during the most recent H1N1 influenza pandemic, there was a significant increase in ICU admissions and mortality among diabetes patients [45,46]. According to reports, pulmonary dysfunction and intensified inflammation are the mechanisms that relate diabetes to increased mortality and shorter survival times in diabetic individuals. Aggressive glycosylation, which causes an excess of advanced glycation end product formation has been linked to the hyperglycaemia brought on by diabetes. It was thought that abnormal glycosylation was connected to immunoglobulin malfunction [39]. Another study showed that prolonged NK cell dysfunction caused by an increased IFN-α response is linked to an unfavorable disease course, supporting NK cells’ role in the immunopathogenesis of COVID-19. [43]

Another study found that decreased cell proliferation induces an adaptive-like NK cell phenotype, which has an early prognostic value for higher TGF and IFN levels in COVID-19 infection, which is related with disease severity. Increased TGF and IFN levels, as well as disease severity, were associated with the accumulation of adaptive-like FcR/low NK cells in COVID-19 patients. One of the key future initiatives will be to analyse these NK phenotypes in the DM and non-DM population to study the association between these unique phenotypes and protections in people with diabetes and COVID-19 [47].

Among patients who developed severe disease, there was a substantial difference between the two groups; diabetics were more likely to develop renal failure and sepsis than non-diabetics. Diabetes patients are predisposed to developing infections of varying severity due to underlying metabolic changes, chronic inflammation, and impaired immunity [48,49]. A more recent study using multiomics showed that in contrast to the expected distinction between mild and moderate infections, the plasma multiomic profiles revealed striking similarities between moderate and severe cases of COVID-19. Changes in lipid, amino acid, and xenobiotic metabolism, as well as a noticeable increase in inflammatory cytokines, characterise this dramatic transformation [50].

The loss of particular classes of metabolites and metabolic processes coincides with an increase in inflammatory signalling, which also showed the significant transition between mild and moderate illness. Many atypical immune cell morphologies occur in this stressed plasma environment during mild disease and become more pronounced with worsening disease. This immune response axis independently coincides with substantial plasma composition alterations, clinical blood coagulation measures, and the abrupt switch from mild to moderate illness [50].

In another report, single cell multiomic analysis revealed a strong interaction between plasma metabolites and metabolic reprogramming networks specific to different cell types that are related to illness severity and may predict survival. It also revealed that a small, metabolically hyperactive fraction of CD8+ T cells, possibly exclusive to the SARS-CoV-2 virus, shows increasing metabolic activity as the disease’s severity increases [51].

The metabolic profile of people with more severe disease was generally more activated, but the level of activation varied by cell type. The significant antiviral activities of CD8+ T cells and B cells were compatible with their elevated metabolic activity, whereas CD4+ T cells, natural killer (NK) cells, and monocytes each showed a somewhat lower elevation. Two distinct monocyte subpopulations have been identified, with inflammatory monocytes increasing in quantity and metabolic activity per cell, while non-classical monocytes act in the other direction [51]. These data suggest that COVID-19 is accompanied with metabolic reprogramming of immune cells and shows that each major type of immune cell has a unique metabolic profile [51]. Examining metabolic activity at the resolution of specific cell types in diabetics with COVID-19 may give insights regarding disease severity and outcome and aid in future research directions.

Understanding immunological responses in COVID-19 patients is crucial for determining the efficacy of therapies, predicting illness prognosis, and comprehending the reported variation in disease severity [49].

A recent study found protective and harmful gene modules that defined unique trajectories associated with moderate versus severe results. The authors argue that despite heterogeneity and regardless of the infecting virus, it is important to identify host response modules since doing so could lead to new intervention options, including diagnostics for identifying patients who are more likely to experience severe consequences [52].

Microvascular complications in diabetes patients include neuropathies and end-stage renal disease [43]. In our study also there was an increase in the risk of acute renal failure in diabetics when compared to non-diabetics, which is a significant finding. Between the diabetic cohort and the non-diabetic cohort, mortality was significantly higher (35.18% vs. 20.6%). Similar to our report, another study found that diabetic COVID-19 patients had a mortality rate of 35.4% while non-diabetic COVID-19 patients had a mortality rate of 20.3% [22]. Post-COVID-19 acute complications are a developing worldwide health issue; type 2 diabetes is one of the potential risk factors. A multiomic analysis of 309 COVID-19 patients suggested that patients prone to post-acute sequelae of COVID-19 may be predicted early in the course of the illness. Immunological connections between post-acute sequelae of COVID-19 infection weaken over time, resulting in different immunological states throughout convalescence. Hence identification of indicators that may indicate long-term disease by comparing patient symptoms with in-depth profiling of blood cells and plasma components throughout COVID-19 infection is needed. [53].

Patients with type 1 and type 2 diabetes mellitus have a high risk of a bad prognosis from COVID-19, and immunisation should be prioritised in this population. However, future studies must address numerous outstanding concerns pertaining to COVID-19 immunisation [54,55]. Clinicians can minimise the burden by advising, reassuring, and supporting older persons with diabetes during the time of epidemics. Providing adequate nourishment through frequent meals and preventing weight loss are more important than optimising the diet at such times [56].

Hyperglycaemic disorders and other coexisting illnesses are made worse by inappropriate medication therapy, which in turn increases morbidity and death. It has been noted that corticosteroids, antivirals, and immunisation raise blood glucose. Alternately, certain biologics, anti-infectives, and antiparasitic medications may decrease blood glucose. In patients with uncontrolled blood glucose who are at risk for diabetes complications, this information may offer recommendations for alternate medications. The healthcare team’s risk/benefit analysis should be applied to this patient population for COVID-19 treatment and prevention recommendations [57]. In our study, we did not fully evaluate the confounding factors and their impact on the disease outcomes which is a limitation in this study.

## 5. Conclusions

Diabetes mellitus enhances the COVID-19 mortality risk and is associated with a particularly severe illness course. In addition, people with diabetes frequently suffer from comorbidities that affect clinical outcomes. COVID-19 influences the pathophysiology of diabetes significantly. In the present study, people with diabetes are more likely than patients without diabetes to develop severe symptoms and problems due to COVID-19. Patients with diabetes were considerably more likely to require intensive care unit (ICU) admission and mechanical breathing, both of which contributed to an increased risk of death. Diabetes is one of the high risk factors for COVID-19, which is associated with a high rate of morbidity and mortality, and hence the need for prompt and adequate care.

## Figures and Tables

**Table 1:** Demographic and clinical characteristics of diabetic and non-diabetic COVID-19 patients.

Variables	Values	Diabetics	Non-Diabetics	*p*-Value
Characteristic				
Number of patients	All (541)	108 (19.96%)	433 (80.04%)	-
Female, *n* (%)	252 (47.01%)	47 (8.77%)	205 (38.25%)	0.4509
Smoker, *n* (%)	44 (11.11%)	8 (2.02%)	36 (9.09%)	0.5684
Age, n (mean, SD)	All (535)	108 (60.3, 14.23)	427 (37.4, 18.8)	<0.0001 *
BMI, n (mean, SD)	All (372)	94 (30.4, 6.17)	278 (27.4, 7.5)	0.0002 *
Signs and Symptoms: n (%), *p*-value from Fisher test for association				
Fever	Yes	53 (50.48%)	108 (32.05%)	0.000 * (OR = 2.16)
	No	52 (49.52%)	229 (67.95%)	
Sore throat	Yes	28 (31.82%)	119 (38.02%)	0.317
	No	60 (68.18%)	194 (61.98%)	
Runny nose	Yes	12 (13.95%)	63 (21.00%)	0.165
	No	74 (86.05%)	237 (79.00%)	
Myalgia Fatigue	Yes	31 (37.35%)	90 (29.32%)	0.181
	No	52 (62.65%)	217 (70.68%)	
Anorexia	Yes	16 (19.51%)	20 (6.87%)	0.002 * (OR = 3.29)
	No	66 (80.49%)	271 (93.13%)	
URTI	Yes	17 (20.48%)	48 (16.00%)	0.326
	No	66 (79.52%)	252 (84.00%)	
Dry cough	Yes	47 (47.96%)	115 (35.06%)	0.024 * (OR = 1.71)
	No	51 (52.04%)	213 (64.94%)	
Productive cough	Yes	27 (27.84%)	45 (14.24%)	0.003 * (OR = 2.32)
	No	70 (72.16%)	271 (85.76%)	
Shortness of breath	Yes	36 (35.29%)	65 (19.82%)	0.002 * (OR = 2.21)
	No	66 (64.71%)	263 (80.18%)	
Chest pain	Yes	16 (16.49%)	23 (7.32%)	0.010 * (OR = 2.50)
	No	81 (83.51%)	291 (92.68%)	
Haemoptysis	Yes	4 (4.60%)	2 (0.65%)	0.023 * (OR = 7.37)
	No	83 (95.40%)	306 (99.35%)	
Headache	Yes	20 (21.05%)	89 (28.53%)	0.185
	No	75 (78.95%)	223 (71.47%)	
Confusion	Yes	6 (6.98%)	1 (0.32%)	0.000 * (OR = 23.48)
	No	80 (93.02%)	313 (99.68%)	
Loss of Consciousness	Yes	4 (4.30%)	2 (0.62%)	0.024 * (OR = 7.17)
	No	89 (95.70%)	319 (99.38%)	
Seizure	Yes	1 (1.10%)	1 (0.32%)	0.397
	No	90 (98.90%)	315 (99.68%)	
Nausea	Yes	15 (16.30%)	32 (10.22%)	0.137
	No	77 (83.70%)	281 (89.78%)	
Vomiting	Yes	16 (16.67%)	27 (8.68%)	0.035 * (OR = 2.1037)
	No	80 (83.33%)	284 (91.32%)	
Abdominal pain	Yes	9 (9.38%)	25 (8.09%)	0.676
	No	87 (90.63%)	284 (91.91%)	
Diarrhoea	Yes	25 (25.77%)	46 (14.29%)	0.012 * (OR = 2.0833)
	No	72 (74.23%)	276 (85.71%)	
Jaundice	Yes	1 (1.25%)	0 (0.00%)	0.214
	No	79 (98.75%)	293 (100.0%)	

* statistically significant.

**Table 2:** Laboratory variables in diabetic and non-diabetic COVID-19 patients.

Variables (Unit)	Diabetics (n = 108) N (Min, Max), (Mean, SD)	Non-Diabetics (n = 434) N (Min, Max), (Mean, SD)	Wilcoxon Statistics	Z	*p*-Value
Haemoglobin (g/L)	93(8.41, 169), (122.77, 25.13)	269(4.25, 173), (132.9, 22.3)	114,838.0	−8.2707	<0.0001 *
Platelet (cell × 10^9^/L)	94(54, 596), (207.79, 84.37)	269(33, 965), (222.94, 82.04)	149,277.0	−0.2375	0.4061
Haematocrit (L/L)	94(0.194, 0.527), (0.38, 0.07)	269(0.043, 436), (2.03, 26.56)	116,667.5	−8.1116	<0.0001 *
PT (Seconds)	83(12.6, 74.7), (16.56, 7.36)	205(12.1, 51.1), (15.8, 4.4)	82,277.00	2.3664	0.0090 *
PTT (Seconds)	83(27.9, 69.2), (39.51, 8.34)	202(1, 68), (38.46, 6.49)	79,134.50	1.3507	0.0884 **
INR	83(0.9, 5.7), (1.22, 0.57)	202(0.9, 42.5), (1.56, 3.98)	81,403.50	1.8947	0.0291 *
Urea (mmol/L)	93(1.5, 73), (9.82, 10.43)	264(0.8, 44), (4.56, 3.93)	200,326.0	12.6036	<0.0001 *
Creatinine (μmol/L)	93(26, 707), (131.49, 126.63)	265(5.2, 768), (79.07, 73.85)	185,319.5	8.6475	<0.0001 *
Sodium (mmol/L)	92(119, 161), (138.02, 6.92)	264(123, 154), (139.45, 3.71)	133,625.0	−3.7126	0.0001 *
Bicarbonate (mmol/L)	76(2.21, 32.1), (21.28, 4.56)	218(14, 105), (23.02, 6.3)	92,179.50	−2.0540	0.0200 *
Glucose (mmol/L)	77(1.3, 25), (11.03, 4.7)	158(3.2, 19.1), (5.82, 1.75)	88,909.00	14.0437	<0.0001 *
Magnesium (mmol/L)	93(0.54, 1.62), (0.81, 0.17)	250(0.46, 83, 1.15), (5.2)	132,511.5	0.0287	0.4885
Calcium (mmol/L)	89(1.48, 92), (3.17, 9.52)	247(1.52, 107), (5.69, 18.12)	97,626.50	−7.3793	<0.0001 *
Phosphate (mmol/L)	87(0.33, 2.8), (1.11, 0.34)	238(0.52, 2.39), (1.15, 0.31)	116,150.5	−1.0748	0.1412
ALT (U/L)	91(6, 411.8), (39.24, 49.11)	255(5, 396), (31.67, 36.63)	141,143.0	2.8275	0.0023 *
AST (U/L)	91(12.7, 1263.8), (53.58, 132.25)	254(3.7, 228), (30.5, 21.59)	147,987.5	4.6573	<0.0001 *
Alkaline Phosphate (U/L)	91(30, 451), (87.08, 57.19)	261(0.13, 968), (97.78, 93.48)	152,252.5	1.5377	0.0621 **
GGT (IU/L)	13(8, 698), (137.92, 187.1)	8(6, 891), (152.75, 301.17)	5303.000	0.8806	0.1893
pH	53(5.5, 8.5), (7.34, 0.41)	99(5.5, 8.35), (7.25, 0.5)	25,501.00	−0.2941	0.3844
PCO2 (K Pascal)	51(2.6, 80.5), (7.15, 11.76)	86(3.1, 43.2), (6.05, 4.63)	23,944.50	−0.3361	0.3684
PO2 (K Pascal)	51(2.9, 52.8), (9.6, 10.2)	86(2.2, 67.6, (6.97, 7.83)	26,673.50	2.7334	0.0031 *
PO2/FiO2 (mmHg)	19(0.22, 325), (129.88, 84.66)	22(0.21, 535.76), (98.42,116.96)	5290.500	−1.5662	0.0587 **
Lactate (mmol/L)	47 (0.6, 6.6), (1.8, 0.95)	83(0.6, 6.5), (1.51, 0.92)	13,214.50	1.8228	0.0342 *
Serum ferritin (μg/L)	43(18.4, 8616), (928.42, 1505.58)	126(4.2, 9838), (365.33, 923.99)	4800.000	4.1311	<0.0001 *
Potassium (mmol/L)	91(2.3, 7.1), (4.33, 0.76)	261(3.2, 5.9), (4.14, 0.45)	157,338.5	2.5077	0.0061 *
Bilirubin (μmol/L)	89 (2, 38.2), (8.81, 6.75)	249(2, 78), (7.12, 6.22)	104,213.0	2.9958	0.0014 *
CK (U/L)	81 (24, 7268), (377.42, 1012.92)	176(6, 1353), (134.02, 152.79)	64,945.50	2.1222	0.0169 *
Troponin (ng/L)	81(2, 541), (33.51, 68.75)	188(2, 305), (9.91, 24.88)	75,626.00	10.0061	<0.0001 *
Specific Gravity	44(1.005, 1.041), (1.02, 0.01)	123(1.003, 1.051), (1.02, 0.01)	3984.500	1.0587	0.1449

* Significant at 0.05, ** Significant at 0.10.

**Table 3:** Complication in diabetic and non-diabetic COVID-19 patient.

Complications		Diabetic		Non-Diabetic		Chi-Seq	*p*-Value
		n	%	n	%		
Patient admitted to ICU	No	32	11.2	167	58.6	54.35	0.000
	Yes	51	17.9	35	12.3		
Patient received mechanical ventilation/intubation	No	53	18.6	185	64.9	32.84	0.000
	Yes	30	10.5	17	6.0		
Patient received ECMO	No	83	29.1	201	70.5	0.41	0.521
	Yes	0	0.0	1	0.4		
Coma	No	82	28.8	201	70.5	0.425	0.514
	Yes	1	0.4	1	0.4		
Encephalitis	No	82	28.8	201	70.5	0.425	0.514
	Yes	1	0.4	1	0.4		
Renal failure	No	73	25.6	196	68.8	9.15	0.002
	Yes	10	3.5	6	2.1		
Seizure	No	82	28.8	199	69.8	0.033	0.855
	Yes	1	0.4	3	1.1		
Sepsis	No	72	25.3	196	68.8	11.1	0.000
	Yes	11	3.9	6	2.1		
Symptoms Resolved	No	37	13.0	59	20.7	6.22	0.013
	Yes	46	16.1%	143	50.2%		
Cured	No	38	13.3%	43	15.1%	17.35	0.001
	Yes	45	15.8%	159	55.8%		

## Data Availability

The data that support the findings of this study are available from King Faisal Specialist Hospital and Research Centre Riyadh and Jeddah, but restrictions apply to the availability of these data, which were used under license for the current study and therefore are not publicly available. Data are, however, available from the authors upon reasonable request and with permission of King Faisal Specialist Hospital and Research Centre Riyadh and Jeddah. The resources generated during and/or analysed during the current study are available from the corresponding author upon reasonable request.
